# Eribulin-induced radiation recall dermatitis: a case report and brief review of the literature

**DOI:** 10.3332/ecancer.2020.1006

**Published:** 2020-01-23

**Authors:** William T Tran, Carolina Ibáñez, Mauricio P Pinto, Cesar Sanchez, Gregory J Czarnota, Tomas Merino

**Affiliations:** 1 Department of Radiation Oncology, Sunnybrook Health Sciences Centre, Toronto ON M4N 3M5, Canada; 2 Department of Radiation Oncology, Faculty of Medicine, University of Toronto, Toronto ON M5S, Canada; 3 Departamento de Hemato-Oncologia, Pontificia Universidad Catolica de Chile, Santiago 8330032, Chile

**Keywords:** radiation recall, eribulin, breast cancer

## Abstract

**Background:**

Radiation recall (RR) is a fairly uncommon and unpredictable phenomenon caused by an acute inflammatory reaction in a previously irradiated area. Several antineoplastic drugs have been previously associated with RR reactions including anthracyclines and taxanes like docetaxel, paclitaxel or antimetabolites.

**Case presentation:**

Here we report for the first time a case of a recall reaction to Eribulin mesylate, a novel chemotherapeutic compound that affects microtubule polymerisation, approved for the treatment of metastatic or locally advanced breast cancers (BCs). We present the case of a 61-year-old female BC patient originally diagnosed with bilateral BC with metastatic disease that went through several lines of chemotherapy and radiation therapy (RT); RR reaction was observed following Eribulin treatment and sequential palliative RT.

**Conclusion:**

This case report raises awareness about these fairly rare phenomena when prescribing Eribulin, or any new chemotherapeutic after RT to prevent and treat as early as possible to avoid further patient complications.

## Background

Radiation recall dermatitis (RRD) is an acute inflammation that develops following exposure to a radiation field [1]. This may occur weeks, months or even years after radiation therapy (RT); RRD typically occurs with the first dose of the chemotherapy agent or combination but may be a long interval between the administration of the causative agent and the appearance of RRD. Most cases occur after the administration of chemotherapeutics like anthracyclines or taxanes [2, 3]. The main symptoms are mild rash, erythema (which may be painful), pruritus, swelling, desquamation and maculopapular eruptions and ulceration. Stem cells deficiency and dysfunction in the irradiated area have been proposed as mechanisms for developing RRD. Indeed, some studies have speculated that stem cells may have an amplified reactivity to radiation, thereby RRD works as a ‘remembered’ biological response from a subsequent drug exposure [1, 2]. Others have suggested that RRD is similar to fixed drug eruptions [3, 4]. Studies demonstrate that radiation potentiates the response to systemically administered agents via a mechanism not fully understood; however, a lowered inflammation threshold after RT has been postulated [3]. Another plausible mechanism is the secretion of inflammatory cytokines stimulated by drugs and secondary to RT [1]. In fact, studies have demonstrated ballooning degeneration, necrosis and inflammatory infiltration in RRD [4].

Significant improvements in the efficacy of chemotherapy for breast cancer (BC) patients in recent years have resulted in better survival rates, generating the need for continued patient care over several years and increasing the chance of patients to receive multiple rounds of chemotherapy after RT treatments. In this context, Eribulin is commonly indicated for disease management and here we describe a case study that examines the association between Eribulin and radiation recall (RR) reaction in a BC female patient who developed RRD secondary to administration of Eribulin.

## Case presentation and discussion

The patient was a 61-year-old woman with bilateral breast masses detected during a routine mammography screening. Right side mammography revealed an ill-defined mass of 3.8 cm × 3.8 cm × 5 cm in the lower-outer quadrant approximately at 5 and 7 o’clock. The left breast showed an ill-defined mass in the outer-upper quadrant that measured 1.7 cm × 1.6 cm × 1.9 cm. A core biopsy showed bilateral invasive ductal carcinoma. The right-side mass was grade 2 (G2), oestrogen receptor (ER)+, progesterone receptor (PR) low and Human Epidermal growth factor Receptor type-2 (HER2)/neu+ (by Fluorescence In Situ Hybridisation). No lymphovascular invasion (LVI). The left mass was grade 3 (G3), LVI- and triple-negative. Computed Tomography (CT) reported bilateral breast malignancies with suspected left internal mammary lymph node metastasis and borderline left axillary lymph nodes. There were non-specific right axillary lymph nodes. Furthermore, no evidence of pulmonary parenchyma or osseous metastatic disease on the thorax was found. However, lytic bone metastases were observed in the pelvis. In the right acetabular roof, there was a lytic lesion measuring 2.9 cm. Sacral and right supra-acetabular metastases are also seen on the bone scan.

Initial patient therapy was weekly Paclitaxel plus Herceptin (H) and Pamidronate for her bony metastasis. After 6 months, the patient’s disease progressed in the breast, liver and bone; Vinorelbine plus Herceptin was then given as second-line with no response. Next, treatment was subsequently switched to Lapatinib plus Capecitabine, and then weekly doxorubicin plus paclitaxel but these two regimens also failed to halt disease progression. Then, a multidisciplinary approach utilised RT to the left breast, supraclavicular area and the internal mammary chain (IMC). The irradiation fields used a standard 4-field technique and an electron patch for the IMC. The patient received 50 Gy in 25 fractions to this area and a 16 Gy boost in eight fractions targeting the macroscopic disease. However, she displayed disease progression in the right lung and liver and received stereotactic body radiotherapy (SBRT) to the lung. The dose regimen to the lung masses was 48 Gy in four fractions and 60 Gy in three fractions for the liver.

Three months later SBRT patient had progression on the left supraclavicular area with axillary and subpectoral lymph nodes. The area was re-irradiated 10 months after the previous RT to a dose of 65 Gy in 50 fractions BID. Again, the patient showed signs of progression in the lung (new lesions) and bones (acetabulum and rib). Palliative RT to the pelvis and rib 20 Gy in five fractions was applied to these areas. The patient then received two cycles of Eribulin. These started 2 weeks after pelvic RT, and 3 months following supraclavicular RT.

**Figure 1.**
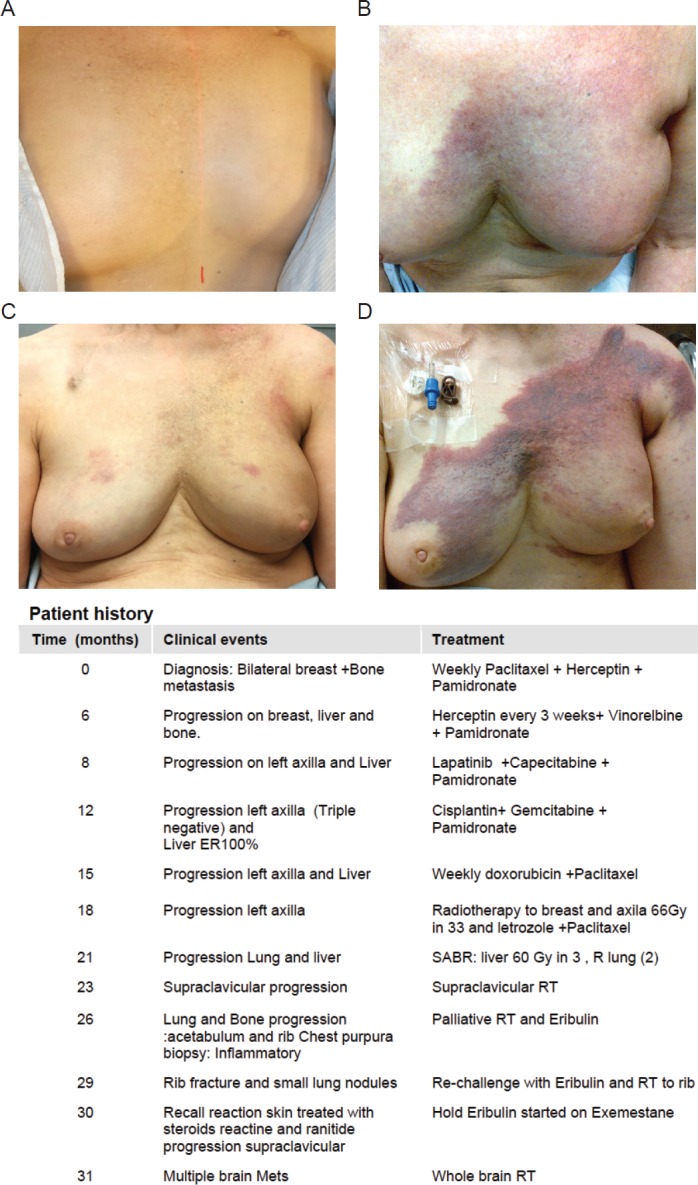
Patient history. (A): Prior to treatment. (B): Acute radio dermatitis following re-irradiation. (C): Patient recovery, 6 months after re-irradiation, start on Eribulin and initial rash reaction. (D): Significant RR after the re-challenge with Eribulin. The table below shows a detailed patient history.

The patient developed a purpuric rash with brisk erythema and moderate edema over the left breast (CTCAE 4.0; grade 2 [5]) and supraclavicular area after she started Eribulin. Figure 1 shows disease progression and subsequent systemic and RT treatments. A biopsy of the skin was performed to rule out disease progression. The results indicated no metastatic disease to the skin but some inflammatory changes. The patient then discontinued the use of Eribulin for 3-months after developing significant fatigue and skin rash consistent with RR reaction. After this period had a re-challenge with Eribulin and a severe skin recall reaction in the left chest wall and over the supraclavicular area. Again, Eribulin was suspended and the patient was given steroids, cetrazine hydrochloride and ranitidine. Unfortunately, she progressed with multiple brain and a right neck metastases. Then, palliative RT was initiated for brain and right neck metastases both at 20 Gy in five fractions.

As survival rates for BC patients increase, a proportional increase in the number of patients exposed to RT followed by chemotherapy will probably result in an increase of RRD cases. Unfortunately, the incidence, prevalence and etiology of symptoms on RR reaction patients are often difficult to assess given the limited number of cohort studies. An observational study by Kodym [6] reported an 8.8% incidence in 91 patients receiving different chemotherapeutic schemes. Similarly, a study by the American Society of Breast Surgeons reported an 11.5% of RR in the Mammosite Breast Brachytherapy Registry Trial.

Several antineoplastic drugs including gemcitabine, capecitabine, 5-FU, doxorubicin, paclitaxel and docetaxel have been associated with recall reactions. More recently, studies have reported RRD following tamoxifen treatment [7, 8]. Eribulin mesylate is a synthetic analog of a halichondrin B, a naturally large polyether macrolide produced by *Halichondria okadai* [9].

Eribulin induces G2/M cell cycle arrest and apoptosis by binding the (+) end of microtubules sequestering tubulin and impairing microtubule growth [9]. *In vitro*, Eribulin has demonstrated activity in taxane-refractory BC and ovarian cancer cell lines [10]. In patients, Eribulin has demonstrated good tolerance in Phase I studies. Also, two Phase III studies have reported the efficacy of Eribulin. First, the EMBRACE trial involved 762 metastasic BC patients with progressive disease after treatment with anthracycline and taxane and demonstrated an increase in overall survival (OS): 13.1 months against 10.6 in the control group [11]. A second study [12] compared Eribulin against capecitabine in 1102 patients and showed a trend towards better OS: 15.9 months in Eribulin versus 14.5 in control; however, this difference did not reach statistical significance (*p* = 0.056). More recently, a Phase II study recommends Eribulin use as first or second-line for metastatic BC [13].

To our knowledge, this is the first case of recall attributed to Eribulin. The case described emphasises the relevance of being aware of this rare phenomenon when prescribing new chemotherapeutics after RT to prevent and treat as early as possible to avoid further patient complications.

## Conclusion

RR is a fairly uncommon phenomenon; however, the ever more frequent use of multiple lines of chemotherapy in BC in patients has the potential of making this phenomenon a more common problem among patients. Whenever possible, sequencing therapies that include radiotherapy followed by chemotherapy should be spaced in time to avoid recall complications. Recall reactions can be diagnosed based on clinical presentation; however, this must be confirmed by biopsy to rule out inflammatory-driven BC. Regarding clinical management of recall events, the first recommendation is to interrupt the use of suspected causing agents and replace with corticoid treatment until recovery. Then, either switch to a different chemotherapeutic drug or resume the treatment with the same drug and lower the dosage. Eribulin is a novel agent for BC that has been proven effective. However, among its side effects we should include RR reactions. Thereby the community of oncologists should be aware of these risks when using novel therapeutic regimens.

## Ethics approval and consent to participate

Since the patient was deceased at the time this manuscript was prepared, the ethics committee at the Pontificia Universidad Catolica de Chile granted a waiver of consent to participate.

## Consent to publish

A written consent for publication was obtained from the patient.

## Availability of data and materials

All data generated or analysed during this study are included in this published article.

## Conflict of interests

None.

## Funding

This work was supported by FONDECYT Iniciacion #11190071 (to TM).

## Authors’ contributions

All authors contributed to writing and editing this manuscript.
